# Aortic stiffness, pulse pressure and cerebral pulsatility progress despite best medical management: the OXVASC cohort

**DOI:** 10.1161/STROKEAHA.121.035560

**Published:** 2021-12-02

**Authors:** Alastair JS Webb, Amy Lawson, Karolina Wartolowska, Sara Mazzucco, Peter M Rothwell

**Affiliations:** Wolfson Centre for Prevention of Stroke and Dementia, University of Oxford, UK

**Keywords:** Arterial stiffness, Aortic blood pressure, Age, Cerebral pulsatility, Small vessel disease;

## Abstract

**Background and Purpose:**

Increased cerebral arterial pulsatility is associated with cerebral small vessel disease, recurrent stroke and dementia despite best medical treatment. However, no study has identified rates and determinants of progression of arterial stiffness and pulsatility.

**Methods:**

In consecutive patients within 6 weeks of TIA or non-disabling stroke (Oxford Vascular Study), arterial stiffness (PWV) and aortic systolic (aoSBP), diastolic (aoDBP) and pulse pressures (aoPP) were measured by applanation tonometry (Sphygmocor), whilst middle cerebral artery peak (MCA-PSV) and trough (MCA-EDV) flow velocity and Gosling’s pulsatility index (MCA-PI) were measured by transcranial ultrasound (TCD, DWL DopplerBox). Repeat assessments were performed at the 5 year follow-up visit after intensive medical treatment and agreement determined by intraclass correlation coefficients (ICC). Rates of progression and their determinants, stratified by age and sex, were determined by mixed-effect linear models, adjusted for age, sex and cardiovascular risk factors.

**Results:**

In 188 surviving, eligible patients with repeat assessments after a median of 5.8 years. PWV, aoPP and MCA-PI were highly reproducible (ICC 0.71,0.59 and 0.65 respectively), with progression of PWV (2.4%, p<0.0001) and aoPP (3.5%, p<0.0001) but not significantly for MCA-PI overall (0.93, p=0.22). However, PWV increased at a faster rate with increasing age (0.009m/s/yr/yr, p<0.0001), whilst aoPP and MCA-PI increased significantly above the age of 55 (aoPP p<0.0001, MCA-PI p=0.009). Higher aortic SBP and DBP predicted a greater rate of progression of PWV and aoPP, but not MCA-PI, although current MCA-PI was particularly strongly associated with concurrent aoPP (p<0.001).

**Conclusions:**

Arterial pulsatility and aortic stiffness progressed significantly after 55 years of age despite best medical treatment. Progression of stiffness and aortic pulse pressure was determined by high blood pressure, but MCA-PI predominantly reflected current aortic pulse pressure. Treatments targetting cerebral pulsatility may need to principally target aortic stiffness and pulse pressure to have the potential to prevent cerebral small vessel disease.

## List of Abbreviations

AF: Atrial fibrillation
BMI: Body mass index
CBFV: Cerebral blood flow velocity
HRV: Heart rate variability
ICC: Intraclass correlation coefficient
MCA: Middle cerebral artery
MMSE: Mini-mental state examination
NIHR: National Institute of Health Research
NYHA: New York Heart Association
OXVASC: Oxford Vascular Study
PI: Pulsatility index
PWV: Pulse wave velocity
SVD: Small vessel disease
TCD: Transcranial doppler

## Introduction

Small vessel disease (SVD) is associated with acute lacunar stroke,^1^ progressive cognitive decline,^2^ late-onset refractory depression,^3^ functional impairment in daily living^4^ and increased mortality.^5^ White matter hyperintensities are highly prevalent in the population, affecting over half of people over the age of 65 and the majority of people over 85.^6^ However, even patients with advanced imaging changes can remain functionally independent,^6^ providing an opportunity for intervention to prevent progression of SVD and subsequent clinical events. White matter hyperintensities are particularly associated with a history of hypertension and markers of vascular aging,^1^ such as aortic stiffness and pulsatility of blood flow in the aorta and the cerebral circulation.^7^ However, despite suggestive post-hoc analyses of clinical trials,^8, 9^ no dedicated prospective study has identified treatments to reduce progression of cerebral small vessel disease.^10, 11^

Reducing cerebral arterial pulsatility and aortic stiffness, and resulting clinical harms, depends upon understanding the rate of progression and identification of the physiological processes responsible for elevated pulsatility. In cross-sectional analyses, cerebral arterial pulsatility is strongly associated with aortic pulse pressure, a relationship that is partially mediated by arterial stiffness, implying increased transmission of aortic pulse pressure to the brain through stiff vessels.^7, 12^ However, there is no evidence for a temporal relationship between arterial stiffness, aortic pulse pressure and cerebral pulsatility and their progression over time. Furthermore, an accurate estimate of the rate of progression and its determinants is critical for the planning and interpretation of clinical trials that aim to prevent progression of arterial stiffness and pulsatility,^13^ particularly after control of classical cardiovascular risk factors such as hypertension.

We therefore determined the rates of progression of residual arterial stiffness, aortic pulse pressure and cerebral arterial pulsatility and their determinants over 5 years in a population of patients with recent TIA or minor stroke, after optimal control of blood pressure and cardiovascular risk factors according to current guidelines.

## Methods

The data that support the findings of this study are available from Prof Rothwell (peter.rothwell@ndcn.ox.ac.uk) upon reasonable request.

Consecutive, consenting patients with TIA or minor stroke were recruited between September 2010 and September 2019, as part of the Phenotyped Cohort of the Oxford Vascular Study (OXVASC).^14, 15^ Participants were recruited at the OXVASC daily emergency assessment clinic, following a referral from primary care or after attendance at the Emergency Department, usually within 24 hours. Patients were referred after transient neurological symptoms or symptoms consistent with a minor stroke, not requiring direct admission to hospital. The OXVASC population consists of >92,000 individuals registered with about 100 primary-care physicians in Oxfordshire, UK.^16^ All consenting patients underwent a standardised medical history and examination, ECG, blood tests and a stroke protocol MRI brain and contrast-enhanced MRA (or CT-brain and carotid Doppler ultrasound or CT-angiogram), an echocardiogram and 5 day ambulatory cardiac monitor. All patients were assessed by a study physician, reviewed by the senior study neurologist (PMR) and are followed-up face-to-face at 1, 3, 6 and 12 months, and 2, 5 and 10 years. Medication is prescribed according to guidelines, most commonly with dual antiplatelets (aspirin and clopidogrel) for one month and monotherapy thereafter, high dose statins (atorvastatin 80mg) and a combination of perindopril and indapamide, with the addition of amlodipine and other agents as required to reach a target of <130/80, guided by home telemetric blood pressure monitoring for the first month in the majority of participants.

As part of the OXVASC Phenotyped cohort, a routine prospective cardiovascular physiological assessment is performed at the 1 month follow-up visit. Since August 2017, all surviving participants still registered with OXVASC primary-care physicians are eligible to undergo a repeat physiological assessment when attending for their 5 year follow-up visit. Participants undergoing a repeat study as part of an assessment for a recurrent cerebrovascular event more than 2.5 years after their initial physiological assessment could also be included. Participants were excluded if they were under 18 years, cognitively impaired (MMSE<23), pregnant, had autonomic failure, a recent myocardial infarction, unstable angina, heart failure (NYHA 3-4 or ejection fraction <40%) or untreated bilateral carotid stenosis (>70%). OXVASC is approved by the Oxfordshire Research Ethics Committee.

Physiological tests were performed at rest in a quiet, dimly-lit, temperature-controlled room (21-230C). Applanation tonometry (Sphygmocor, AtCor Medical, Sydney, Australia) was used to measure carotid-femoral pulse wave velocity (aortic-PWV), aortic augmentation index and aortic systolic and diastolic blood pressure and pulse pressure (ao-SBP, ao-DBP, ao-PP),^14^ with consistent sites of measurement at baseline and follow-up, as possible. TCD (Doppler Box, Compumedics DWL, Singen, Germany) was performed with a 2MHz probe at the temporal bone window on the same side as carotid applanation, where possible. The waveform envelope was acquired at 100Hz simultaneously with ECG and blood pressure at 200Hz (Finometer, Finapres Medical Systems, The Netherlands), via a Powerlab 8/30 with LabChart Pro software (ADInstruments, USA).^17^ The MCA was insonated at the site of peak velocity closest to 50mm, or if this was not adequate, at the depth giving the optimal waveform, excluding patients with velocity transitions indicative of a focal MCA stenosis. All waveforms were visually inspected and beats corrupted by artefact were excluded. Absolute peak, trough and mean cerebral blood flow velocoty (CBFV) was calculated as the average of the remaining beats during a 15 second window, from the envelope of the spectrum. Where reported, mean BP was calculated as DBP +1/3*PP. MCA pulsatility was calculated as Gosling’s pulsatility index (MCA-PI= (systolic CBFV-diastolic CBFV) / mean CBFV). Rate of change in pulse wave velocity and aortic and cerebral haemodynamic indices were determined as the absolute difference or the percentage change from baseline, divided by the number of years between assessments.

Consistency in measures between baseline and follow-up were determined by intraclass correlation coefficients and linear regression, and visually by Bland-Altman plots. Significant changes in indices between baseline and follow-up were assessed by paired t-test. Rates of progression of measures of arterial stiffness, aortic blood pressure and cerebral blood flow velocity were determined by linear mixed effect modelling, with autoregressive covariance structure to account for repeated measures. Analyses were stratified by age and gender, with rates of progression determined by the interaction with the time interval between visits, as continuous indices and stratified by age group (<55, 55-65, 65-75, >75). Potential determinants of absolute values and rates of progression were assessed, unadjusted and adjusted for age, gender and cardiovascular risk factors (smoking, dyslipidaemia, diabetes, hypertension). Results are reported according to the STROBE reporting guideline.^18^

Analyses were performed in R and Matlab r2018.

## Results

188 of 310 eligible patients were seen for follow up at a median of 5.8 years after the initial assessment (supplemental figure I). Of the included patients, 150 had arterial stiffness assessments at baseline and follow-up, whilst 139 had TCD performed on both occasions. Demographic characteristics were similar between patients undergoing arterial stiffness and TCD measures (Table 1).

**Figure 1:**
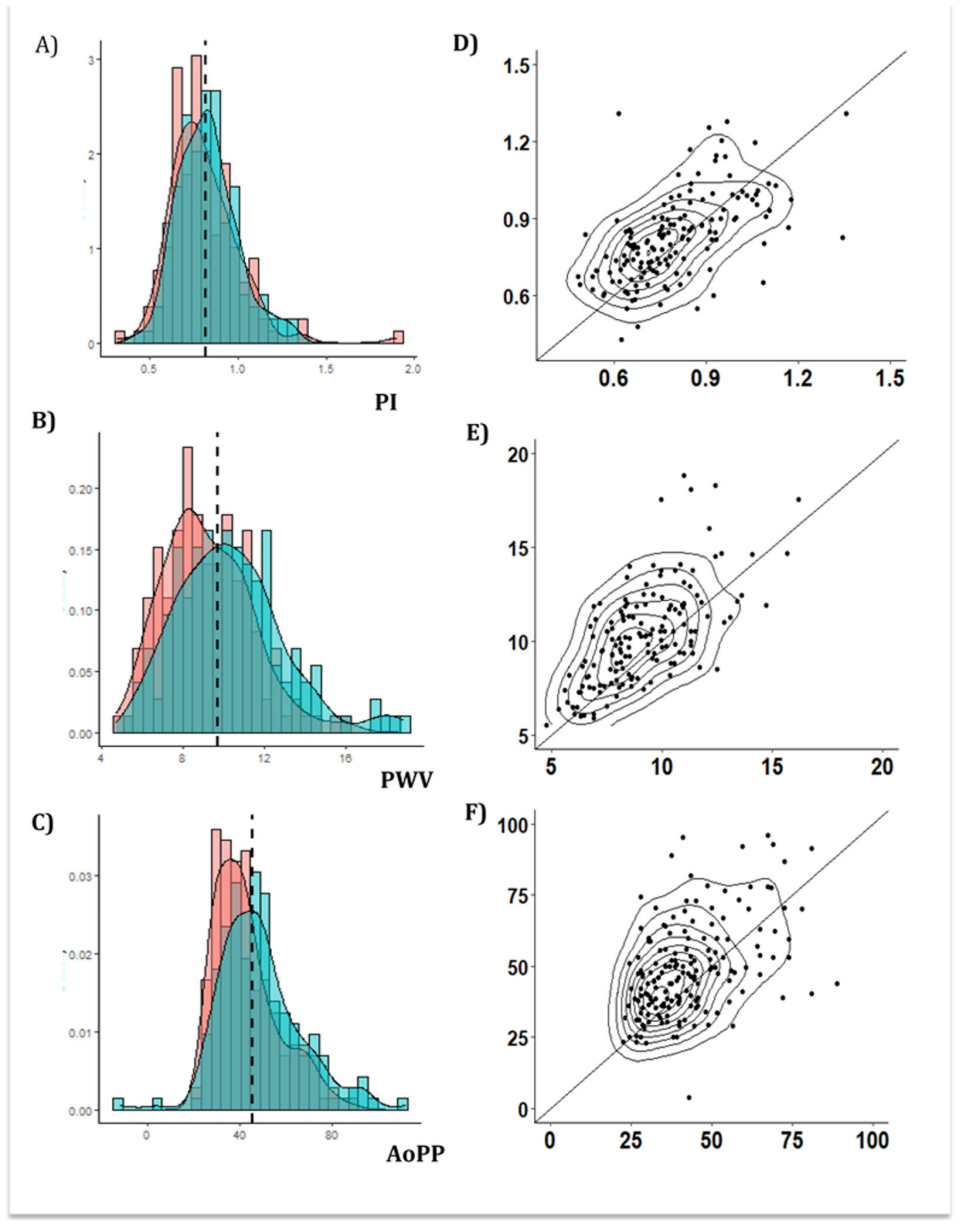
Distribution and reproducibility of middle cerebral artery pulsatility index (PI), aortic pulse wave velocity (PWV) and aortic pulse pressure (AoPP). Panels A-C show the distributions for PI, PWV and AoPP respectively, for baseline vs follow-up. Panels D-F show the scatter plots for baseline vs follow, including a line of unity.

Arterial stiffness (PWV), aortic pulse pressure and cerebral arterial pulsatility were all highly reproducible within individuals over the five years of follow-up (table 2, supplemental figure II), with PWV the most reproducible index. Absolute indices of aortic blood pressure or cerebral blood flow velocity were also signficantly reproducible, but less so than pulsatility or arterial stiffness measures, with weaker correlations between baseline and follow-up values (table 2, figure 1).

There were significant increases in arterial stiffness and aortic pulse pressure between baseline and follow-up, with the greatest annual increase in aortic pulse pressure (3.5% per year). Cerebral arterial pulsatility increased non-significantly across all patients (0.93%, table 2), but was affected by a single outlier with a fall in PI of more than 4 standard deviations from the mean. In a post-hoc sensitivity analysis, exclusion of this patient demonstrated significant overall increase (absolute change per annum 0.005, 1.02%, p=0.04). The increase in aortic pulse pressure reflected a significant fall in aortic DBP (table 2, supplemental figure 3), with a rise in aortic SBP (p=0.01), but both cerebral peak and trough flow velocities fell with time (table 2).

**Figure 2:**
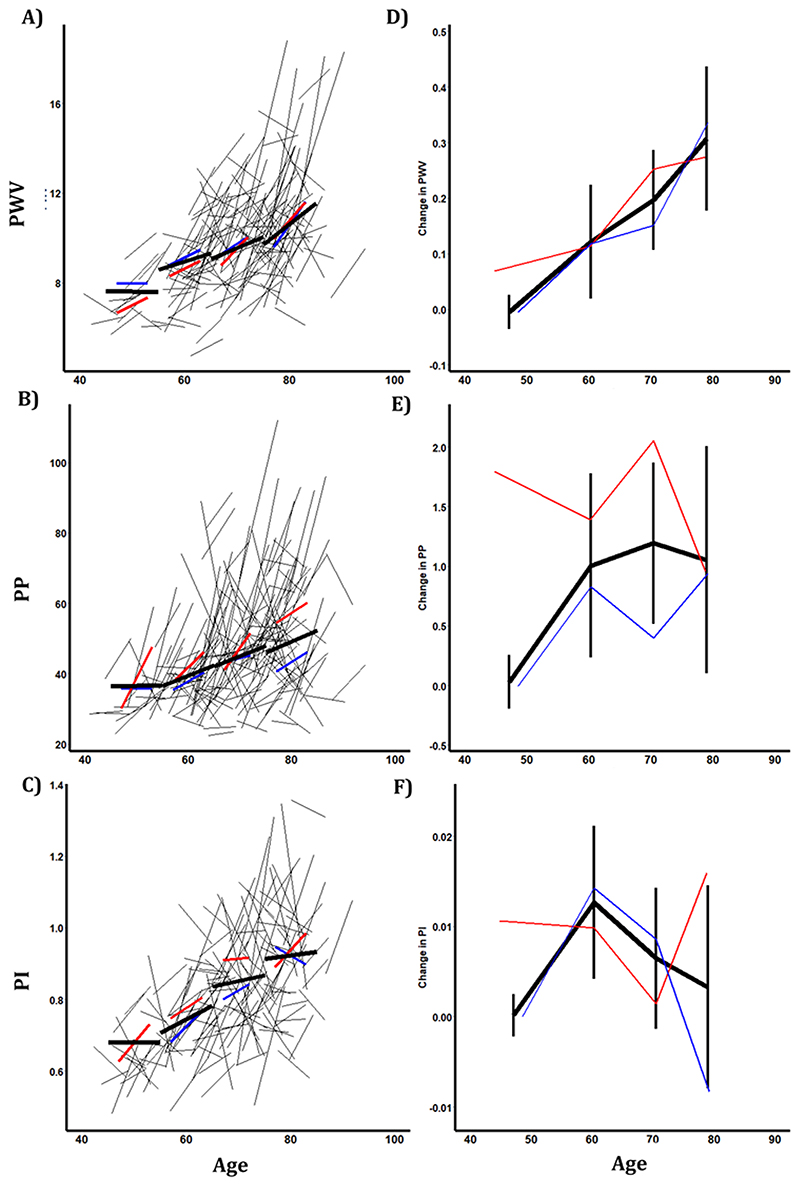
Progression of aortic blood pressure and cerebral blood flow velocity by age and gender. Panels A-C show individual changes during follow-up, and summary estimates within age groups (<55, 55-65,65-75, >75), for all patients (black), for men (blue) and women (red). Panels D-F show the average rate of progression within each age group, stratified by age and gender.

Carotid-femoral PWV was higher at older ages, with no significant yearly increase in patients below the age of 55, but PWV increased at a greater annual rate with increasing age (figure 2, table 1). Although pulse wave velocity was greater in men at each age, the rate of increase in PWV with age was similar in men and women (figure 1). Similarly, both aortic pulse pressure and cerebral arterial pulsatility increased at a greater rate above the age of 55 with no significant increase before 55 years (>55 vs < 55: aoPP p=0, MCA-PI p=0.009), although the rate of change of aoPP or MCA-PP above 55 years was not consistent across age groups (figure 2).

Aortic pulse pressure was significantly greater in women than men and increased at a faster rate (Table 3), whilst MCA-PI was non-significantly greater in women than men with no difference in the rate of increase (Figure 1, Table 3). The rise in aortic and cerebral pulsatility at older ages was paralleled by a higher aortic SBP with increasing age, with a higher rate of increase at older ages. In contrast, PSV and EDV were lower at older ages, and were lower in men compared to women, whilst the rate of change in PSV and EDV was similar across ages (supplemental table I, supplemental figure II).

Elevated aortic SBP, DBP and pulse pressure were all associated with a greater rate of progression of PWV (Table 4), but cross-sectionally, higher PWV was associated with a lower concurrent DBP reflecting the decrease in DBP with time and age. After adjustment for heart rate and other aortic blood pressure values, only a higher aortic mean pressure was associated with a greater rate of increase in PWV with time (p=0.048). Similarly, higher aortic SBP and DBP, elevated heart rate and a greater cerebral pulsatility were associated with a greater rate of increase in aortic pulse pressure, with persistent associations with progression of aortic pulse pressure and aortic systolic and diastolic blood pressure, even after adjustment for arterial stiffness and aortic blood pressure (p=0.0001 for both).

In contrast to aortic measures, a greater rate of progression of MCA-PI was only associated with increased age and female gender (table 3) and a lower cerebral end-diastolic velocity (table 4), with no association between blood pressure level or arterial stiffness with progression of MCA-PI before or after adjustment for age, gender and cardiovascular risk factors. However, there were strong cross-sectional associations between MCA-PI and a concurrent high aortic SBP, low aortic DBP and high aortic PP.

## Discussion

This is the first study of progression of aortic stiffness, aortic pulse pressure and cerebral arterial pulsatility in a single patient cohort, following good blood pressure control. Over five years, aortic stiffness and aortic systolic pressure rose whilst aortic diastolic pressure fell, with a resulting increase in aortic pulse pressure. Cerebral pulsatility rose above the age of 55, reflecting a fall in end-diastolic velocity with no rise in peak systolic velocity. The rate of rise in PWV and aortic pulse pressure was associated with age and elevated aortic blood pressure, but only age, female gender and a low end-diastolic flow velocity predicted an increased rate of rise in MCA-PI, although current MCA-PI level was strongly associated with concurrent aortic pulse pressure.

In previous cross-sectional analyses in this population,^7, 17^ cerebral small vessel disease was associated with cerebral arterial pulsatility, which was principally associated with aortic stiffness and transmission of increased aortic pulse pressure to the brain. Furthermore, there were similar distributions of aortic stiffness, aortic pulse pressure and cerebral arterial pulsatility by age and sex, and the cross-sectional relationship between aortic pulse pressure and cerebral arterial pulsatility was partially mediated by arterial stiffness.^19^ This longitudinal follow-up study confirms the strong concurrent association between aortic pulse pressure, aortic stiffness and cerebral arterial pulsatility, and further demonstrates that aortic blood pressure level predicts a greater increase in the rate of progression of aortic stiffness and pulsatility. As such, although an acute reduction in aortic pulse pressure may reduce cerebral arterial pulsatility directly, control of mid-life elevated aortic blood pressure, and diastolic blood pressure in particular, may reduce progression of arterial stiffness and aortic pulse pressure and therefore future cerebral small vessel disease and its sequelae. Furthermore, this population had undergone intensive blood pressure monitoring and treatment, with a minimal difference between treatment at the time of initial assessment and at follow-up five years later,^20^ although there was an increase in clinic SBP between baseline and follow-up, potentially influencing measurement of PWV, although this could reflect a white-coat response especially as home readings were not available. Nonetheles, this study demonstrates that residual aortic blood pressure and pulsatility was still associated with increased cerebral pulsatility despite excellent control of blood pressure and other risk factors, presenting a potentially modifiable risk factor in addition to current standard treatment.

The majority of the progression in arterial stiffness and pulsatility occurred after the age of 55, with steadily increasing rates of progression of arterial stiffness consistent with previous studies in hypertensive populations. Both aortic and cerebral pulsatility were greater above 55 years, but with a limited ongoing increase in the rate of progression thereafter. This apparent transition at approximately 55 years old is consistent with the average age of transition to late-life hypertensive phenotypes,^21^ and the stronger relationship between mid-life elevated diastolic blood pressure with cerebral white matter hyperintensities,^22^ compared to greater SBP and pulsatility above the age of 55. Furthermore, although DBP fell as arterial stiffness increased, a higher aortic DBP was still the strongest driver of progression of PWV, and therefore a subsequent fall in DBP. This indicates the likely critical role for control of mid-life DBP to prevent later progression of aortic stiffness and pulsatility, and resulting cerebral pulsatility.

Although no systemic haemodynamic measures were specifically associated with progression of cerebral pulsatility, current cerebral pulsatility was most strongly associated with concurrent aortic pulse pressure. As such, targetting both excess aortic systolic pressure and diastolic pressure in mid-life whilst not excessively reducing diastolic pressure in late life, may reduce progression of aortic pulse pressure and therefore later-life cerebral arterial pulsatility. This may be possible with peripheral vasodilators that reduce wave reflection from the peripheral circulation. For example, in the CAFE study,^23^ amlodipine reduced aortic systolic pressure to a greater extent than a beta-blocker, whilst cilostazol reduced cerebral pulsatility in the ECLIPSE study.^24^ Currently, isosorbide mononitrate and cilostazol are being assessed in the LACI-1/LACI-2 studies (ISRCTN 14911850),^25^ antihypertensives in the TREAT-SVDs trial (NCT03082014) and sildenafil in the OxHARP trial (NCT03855332).

This study does have some limitations. Firstly, it is relatively small. However, it is still significantly larger than any other available study recording both aortic and cerebral indices, and other studies have not assessed progression of these measures in this patient population.^26, 27^ Furthermore, despite the moderate size, associations were strong and consistent with previous evidence whilst building upon it. Secondly, not all participants underwent both TCD and arterial stiffness measures. This is inevitable in a pragmatic study including all patients with TIA or minor stroke, due to the relatively high incidence of participants with poor bone windows for TCD or, for PWV, significant large artery disease or increased BMI. Thirdly, due to the age and frailty of the population only 50% of the baseline population could be reassessed at 5 years, as many were deceased or had moved out of the area. This may result in underestimation of the degree of progression of age-related variables due to attrition of higher risk patients with greater progression, but any resulting bias is likely to be conservative. Furthermore, the median age of the population was relatively old for a follow-up study (mean age 66 at baseline, 71 at follow-up), supporting the relevance of these results to the population most at risk. Fourthly, patients with AF were included in the analyses as PWV and PI are still relevant in this group, but AF may reduce reliability of assessment of PWV in particular. Finally, without repeat brain imaging, we were unable to assess whether progression of cerebral arterial pulsatility mediates the relationship between arterial stiffness, aortic pulse pressure and progression of cerebral small vessel disease.

Overall, the strong relationship between residual aortic blood pressure after intensive blood pressure treatment and longitudinal progression of arterial stiffness and pulsatility supports the need for further research to determine the temporal relationship with other key physiological mechanisms affected in small vessel disease, such as blood pressure variability, cerebral autoregulation and cerebrovascular reactivity, that represent alternative potential treatment targets. Further trials will then be necessary to determine if modifying cerebral pulsatility or other mechanisms translates to a reduction in progression of cerebral small vessel disease, proceeding to large clinical trials to determine if such interventions translate to meaningful clinical effects.

## Conclusions

Arterial pulsatility and aortic stiffness progressed significantly over the age of 55 in a population of patients with TIA or minor stroke. Progression of aortic measures was most strongly associated with age and elevated aortic blood pressure, whilst only age, sex and low diastolic cerebral blood flow velocity predicted progression of cerebral arterial pulsatility. However, aortic pulse pressure was the strongest association of concurrent cerebral pulsatility, identifying a potentially treatable mechanism to limit progression of cerebral small vessel disease.

## Supplementary Material

Supplemental Combined PDF

## Figures and Tables

**Table 1:** Demographics of patients at presentation with repeat studies after 5 years follow-up. The table shows distribution of patients who underwent pulse wave velocity (PWV) measures or cerebral pulsatility measures (PI) at baseline and follow-up. Results are reported as frequency (percentage) or as mean (standard deviation). The p-value (p) is shown for t-tests or a test of proportions. BMI = body mass index; SBP =systolic blood pressure; DBP = diastolic blood pressure.

	PWV	PI	p
n	150	139	
Age	66 (10.9)	65 (10.9)	0.4
Female	57 (38)	50 (36)	0.81
Hypertension	112 (74.7)	102 (73.4)	0.91
Diabetes	19 (12.7)	13 (9.4)	0.48
Atrial Fibrillation	13 (8.7)	12 (8.6)	1
Dyslipidaemia	116 (77.3)	107 (77)	1
Current Smoker	13 (8.7)	16 (11.5)	0.54
Past Smoker	67 (44.7)	64 (46)	0.91
Medications	-	-	-
Antiplatelet	119 (79.3)	108 (77.7)	0.85
Antihypertensives	109 (72.7)	97 (69.8)	0.68
Statins	111 (74)	103 (74.1)	1
Weight (Kg)	77.6 (14.8)	78.9 (16.2)	0.52
BMI (Kg/m2)	26.6 (4.1)	26.8 (4.6)	0.57
Creatinine	78.4 (19.8)	76.4 (19.2)	0.36
SBP (presentation)	149.3 (24.8)	150.9 (24.8)	0.45
DBP (presentation)	85.3 (13.7)	86.4 (13.7)	0.4
SBP (1 month)	132.8 (17.4)	132.7 (19.0)	0.85
DBP (1 month)	76.8 (11.2)	78.1 (11.5)	0.59

**Table 2:** Reproducibility and progression of arterial stiffness, aortic blood pressure and cerebral blood flow velocity. Results are shown for aortic and cerebral haemodynamic indices, reporting the intraclass correlation coefficient (ICC) and r-squared (r^2^) from linear regression, with the mean values at baseline and the absolute and percentage change at follow-up. P-values (p) are shown for the linear regression and paired t-test. SBP = systolic blood pressure; DBP =diastolic blood pressure; PWV = pulse wave velocity; MCA = middle cerebral artery; PSV = peak systolic velocity; EDV = end-diastolic velocity; PI = pulsatility index; 95%CI = 95 percent confidence interval.

Index	ICC	ICC 95%CI	r^2^	p	Baseline Mean	FU Mean	t-test p	Annual change	Annual change (%)
PWV	0.71	(0.62 - 0.78)	0.42	<0.001	9.18	10.3	<0.001	0.19	2.4
Aortic SBP	0.45	(0.29 - 0.57)	0.10	<0.001	117	122	0.0068	0.80	0.87
Aortic DBP	0.47	(0.32 - 0.59)	0.10	<0.001	74.1	72.2	0.016	-0.31	-0.24
Aortic PP	0.59	(0.47 - 0.68)	0.23	<0.001	42.8	49.4	<0.001	1.1	3.5
MCA PSV	0.59	(0.47 - 0.69)	0.18	<0.001	81.8	80	0.29	-0.36	0.23
MCA EDV	0.70	(0.60 - 0.77)	0.3	<0.001	39.5	37.7	0.017	-0.36	-0.39
MCA PI	0.65	(0.54 - 0.74)	0.24	<0.001	0.808	0.827	0.22	0.0037	0.93

**Table 3:** Associations between demographic characteristics and progression of key indices of arterial stiffness and pulsatility. Results from mixed effect linear models, adjusted for age, gender and cardiovascular risk factors are shown, for absolute level and progression (change) of aortic pulse wave velocity (PWV), aortic pulse pressure (PP) and middle cerebral artery pulsatility index (PI). B = unstandardized beta-coefficient; p= p-value. Beta-coefficients and p-values for change in each index are derived from the interaction term between the index of interest and the time interval between assessments.

Index	PWV		PWV change		PP		PP change		PI		PI change	
	b	p	b	p	b	p	b	p	b	p	b	p
Female	-0.59	0.068	0.18	0.00035	3.4	0.092	1.6	<0.0001	0.024	0.34	0.0078	0.049
Age	0.075	<0.0001	0.009	<0.0001	0.36	<0.0001	0.045	0.00023	0.0067	<0.0001	0.00029	0.038
Hypertension	0.78	0.045	-0.054	0.41	5	0.043	0.022	0.96	0.048	0.092	-0.0081	0.082
Diabetes	0.93	0.071	0.2	0.013	-3	0.35	0.62	0.28	0.049	0.27	0.00036	0.96
Atrial Fibrillation	-1.1	0.081	0.22	0.033	-5.6	0.14	1.2	0.1	-0.096	0.033	0.00098	0.9
Dyslipidaemia	0.3	0.47	-0.13	0.05	-2.9	0.27	0.1	0.83	0.0053	0.86	-0.0023	0.66
Smoking	0.37	0.26	-0.16	0.00021	7.1	0.00084	-0.94	0.002	0.068	0.0068	-0.0082	0.013
Current Smoking	0.045	0.94	0.051	0.62	1.6	0.63	0.29	0.66	-0.0043	0.91	0.0027	0.71
Weight	0.027	0.016	-0.0055	0.00072	0.03	0.67	-0.024	0.028	-0.0011	0.2	0	0.44
BMI	0.078	0.05	-0.0098	0.16	-0.15	0.54	0.036	0.43	-0.0031	0.27	0	0.95
Antihypertensive	0.61	0.11	-0.03	0.64	3.4	0.15	0.35	0.43	0.044	0.11	-0.0078	0.088
Statin	0.33	0.39	-0.087	0.17	-1.2	0.62	0.11	0.81	0.016	0.58	-0.0029	0.55

**Table 4:** Associations between physiological characteristics and progression of key indices of arterial stiffness and pulsatility. Results from mixed effect linear models, adjusted for age, gender and cardiovascular risk factors are shown, for absolute level and progression (change) of aortic pulse wave velocity (PWV), aortic pulse pressure (PP) and middle cerebral artery pulsatility index (PI). Beta-coefficients and p-values for change in each index are derived from the interaction term between the index of interest and the time interval between assessments. b = unstandardized betacoefficient; p= p-value; HRV = heart rate variability; SBP = systolic blood pressure; DBP =diastolic blood pressure; MCA = middle cerebral artery; PSV = peak systolic velocity; EDV = end-diastolic velocity; PI = pulsatility index.

Index	PWV		PWV change		PP		PP change		PI		PI change	
	B	p	b	p	b	p	b	p	b	p	b	p
Heart Rate	-0.004	<0.001	0.0003	0.059	0.014	0.044	0.003	0.034	<0.001	0.0098	0	0.86
HRV	-0.001	0.98	0.0028	0.73	0.084	0.79	-0.062	0.28	-0.008	0.02	0.0002	0.69
PWV					1.9	0.0003	0.059	0.5	0.011	0.079	0	0.96
Ao SBP	0.013	0.13	0.0047	8e-04	0.62	<0.001	0.016	<0.001	0.0014	0.034	0.0001	0.18
Ao DBP	-0.01	0.48	0.011	1e-04	0.1	0.26	0.082	<0.001	-0.003	0.0057	0.0002	0.46
Ao PP	0.031	0.007	0.0038	0.05					0.0044	<0.001	0	0.60
MCA PSV	-0.008	0.37	0	0.77	0.13	0.037	-0.008	0.5	0.0031	<0.001	0	0.18
MCA EDV	-0.021	0.27	-0.005	0.057	-0.003	0.98	-0.051	0.0086	-0.003	0.0068	0	0.014
MCA PI	0.44	0.66	0.44	0.0054	30	<0.001	3.2	0.0023				
