# Aortic Stiffness, Pulse Pressure, and Cerebral Pulsatility Progress Despite Best Medical Management: The OXVASC Cohort

**DOI:** 10.1161/STROKEAHA.121.035560

**Published:** 2021-12-02

**Authors:** Alastair J.S. Webb, Amy Lawson, Karolina Wartolowska, Sara Mazzucco, Peter M. Rothwell

**Affiliations:** Department of Clinical Neurosciences, Wolfson Centre for Prevention of Stroke and Dementia, University of Oxford, United Kingdom.

**Keywords:** arterial pressure, follow-up studies, leukoaraiosis, linear models, vascular stiffness

## Abstract

**Background:**

Increased cerebral arterial pulsatility is associated with cerebral small vessel disease, recurrent stroke, and dementia despite the best medical treatment. However, no study has identified the rates and determinants of progression of arterial stiffness and pulsatility.

**Methods:**

In consecutive patients within 6 weeks of transient ischemic attack or nondisabling stroke (OXVASC [Oxford Vascular Study]), arterial stiffness (pulse wave velocity [PWV]) and aortic systolic, aortic diastolic, and aortic pulse pressures (aoPP) were measured by applanation tonometry (Sphygmocor), while middle cerebral artery (MCA) peak (MCA-PSV) and trough (MCA-EDV) flow velocity and Gosling pulsatility index (PI; MCA-PI) were measured by transcranial ultrasound (transcranial Doppler, DWL Doppler Box). Repeat assessments were performed at the 5-year follow-up visit after intensive medical treatment and agreement determined by intraclass correlation coefficients. Rates of progression and their determinants, stratified by age and sex, were determined by mixed-effects linear models, adjusted for age, sex, and cardiovascular risk factors.

**Results:**

In 188 surviving, eligible patients with repeat assessments after a median of 5.8 years. PWV, aoPP, and MCA-PI were highly reproducible (intraclass correlation coefficients, 0.71, 0.59, and 0.65, respectively), with progression of PWV (2.4%; *P*<0.0001) and aoPP (3.5%; *P*<0.0001) but not significantly for MCA-PI overall (0.93; *P*=0.22). However, PWV increased at a faster rate with increasing age (0.009 m/s per y/y; *P*<0.0001), while aoPP and MCA-PI increased significantly above the age of 55 years (aoPP, *P*<0.0001; MCA-PI, *P*=0.009). Higher aortic systolic blood pressure and diastolic blood pressure predicted a greater rate of progression of PWV and aoPP, but not MCA-PI, although current MCA-PI was particularly strongly associated with concurrent aoPP (*P*<0.001).

**Conclusions:**

Arterial pulsatility and aortic stiffness progressed significantly after 55 years of age despite the best medical treatment. Progression of stiffness and aoPP was determined by high blood pressure, but MCA-PI predominantly reflected current aoPP. Treatments targetting cerebral pulsatility may need to principally target aortic stiffness and pulse pressure to have the potential to prevent cerebral small vessel disease.

Small vessel disease (SVD) is associated with acute lacunar stroke,^1^ progressive cognitive decline,^2^ late-onset refractory depression,^3^ functional impairment in daily living,^4^ and increased mortality.^5^ White matter hyperintensities are highly prevalent in the population, affecting over half of people over the age of 65 years and the majority of people over 85 years of age.^6^ However, even patients with advanced imaging changes can remain functionally independent,^6^ providing an opportunity for intervention to prevent progression of SVD and subsequent clinical events. White matter hyperintensities are particularly associated with a history of hypertension and markers of vascular aging,^1^ such as aortic stiffness and pulsatility of blood flow in the aorta and the cerebral circulation.^7^ However, despite suggestive post hoc analyses of clinical trials,^8,9^ no dedicated prospective study has identified treatments to reduce progression of cerebral SVD.^10,11^

Reducing cerebral arterial pulsatility and aortic stiffness, and resulting clinical harms, depends upon understanding the rate of progression and identification of the physiological processes responsible for elevated pulsatility. In cross-sectional analyses, cerebral arterial pulsatility is strongly associated with aortic pulse pressure (aoPP), a relationship that is partially mediated by arterial stiffness, implying increased transmission of aoPP to the brain through stiff vessels.^7,12^ However, there is no evidence for a temporal relationship between arterial stiffness, aoPP, and cerebral pulsatility and their progression over time. Furthermore, an accurate estimate of the rate of progression and its determinants is critical for the planning and interpretation of clinical trials that aim to prevent progression of arterial stiffness and pulsatility,^13^ particularly after control of classical cardiovascular risk factors such as hypertension.

We, therefore, determined the rates of progression of residual arterial stiffness, aoPP, and cerebral arterial pulsatility and their determinants over 5 years in a population of patients with recent transient ischemic attack or minor stroke, after optimal control of blood pressure and cardiovascular risk factors according to current guidelines.

## Methods

The data that support the findings of this study are available from P.M.R. (peter.rothwell@ndcn.ox.ac.uk) upon reasonable request.

Consecutive, consenting patients with transient ischemic attack or minor stroke were recruited between September 2010 and September 2019, as part of the Phenotyped Cohort of OXVASC (Oxford Vascular Study).^14,15^ Participants were recruited at the OXVASC daily emergency assessment clinic, following a referral from primary care or after attendance at the Emergency Department, usually within 24 hours. Patients were referred after transient neurological symptoms or symptoms consistent with a minor stroke, not requiring direct admission to hospital. The OXVASC population consists of >92 000 individuals registered with about 100 primary care physicians in Oxfordshire, United Kingdom.^16^ All consenting patients underwent a standardized medical history and examination, ECG, blood tests, and a stroke protocol magnetic resonance imaging brain and contrast-enhanced MRA (or computed tomography brain and carotid Doppler ultrasound or computed tomography angiogram), an echocardiogram, and 5 day ambulatory cardiac monitor. All patients were assessed by a study physician, reviewed by the senior study neurologist (P.M.R.), and were followed up face to face at 1, 3, 6, and 12 months and 2, 5, and 10 years. Medication is prescribed according to guidelines, most commonly with dual antiplatelets (aspirin and clopidogrel) for 1 month and monotherapy thereafter, high-dose statins (atorvastatin, 80 mg), and a combination of perindopril and indapamide, with the addition of amlodipine and other agents as required to reach a target of <130/80, guided by home telemetric blood pressure monitoring for the first month in the majority of participants.

As part of the OXVASC phenotyped cohort, a routine prospective cardiovascular physiological assessment is performed at the 1-month follow-up visit. Since August 2017, all surviving participants still registered with OXVASC primary care physicians are eligible to undergo a repeat physiological assessment when attending for their 5-year follow-up visit. Participants undergoing a repeat study as part of an assessment for a recurrent cerebrovascular event >2.5 years after their initial physiological assessment could also be included. Participants were excluded if they were under 18 years of age, cognitively impaired (Mini-Mental State Examination <23), pregnant, and had autonomic failure, a recent myocardial infarction, unstable angina, heart failure (New York Heart Association class 3–4 or ejection fraction <40%), or untreated bilateral carotid stenosis (>70%). OXVASC is approved by the Oxfordshire Research Ethics Committee.

Physiological tests were performed at rest in a quiet, dimly lit, temperature-controlled room (21–23 °C). Applanation tonometry (Sphygmocor; AtCor Medical, Sydney, Australia) was used to measure carotid-femoral pulse wave velocity (PWV; aortic PWV), aortic augmentation index, and aortic systolic blood pressure (aoSBP) and aortic diastolic blood pressure (aoDBP) and aoPP,^14^ with consistent sites of measurement at baseline and follow-up, as possible. Transcranial Doppler (TCD; Doppler Box; Compumedics DWL, Singen, Germany) was performed with a 2-MHz probe at the temporal bone window on the same side as carotid applanation, where possible. The waveform envelope was acquired at 100 Hz simultaneously with ECG and blood pressure at 200 Hz (Finometer; Finapres Medical Systems, the Netherlands), via a Powerlab 8/30 with LabChart Pro software (ADInstruments).^17^ The middle cerebral artery (MCA) was insonated at the site of peak velocity closest to 50 mm, or if this was not adequate, at the depth giving the optimal waveform, excluding patients with velocity transitions indicative of a focal MCA stenosis. All waveforms were visually inspected, and beats corrupted by artifact were excluded. Absolute peak, trough, and mean cerebral blood flow velocity (CBFV) was calculated as the average of the remaining beats during a 15-second window, from the envelope of the spectrum. Where reported, mean BP was calculated as diastolic blood pressure (DBP)+1/3×pulse pressure. MCA pulsatility was calculated as Gosling pulsatility index (PI; MCA-PI=[systolic CBFV–diastolic CBFV]/mean CBFV). Rate of change in PWV and aortic and cerebral hemodynamic indices were determined as the absolute difference or the percentage change from baseline, divided by the number of years between assessments.

Consistency in measures between baseline and follow-up was determined by intraclass correlation coefficients and linear regression and visually by the Bland-Altman plots. Significant changes in indices between baseline and follow-up were assessed by paired *t* test. Rates of progression of measures of arterial stiffness, aortic blood pressure, and CBFV were determined by linear mixed-effects modeling, with autoregressive covariance structure to account for repeated measures. Analyses were stratified by age and sex, with rates of progression determined by the interaction with the time interval between visits, as continuous indices and stratified by age group (<55, 55–65, 65–75, and >75). Potential determinants of absolute values and rates of progression were assessed, unadjusted, and adjusted for age, sex, and cardiovascular risk factors (smoking, dyslipidemia, diabetes, and hypertension). Results are reported according to the STROBE reporting guideline.^18^

Analyses were performed in R and Matlab r2018.

## Results

One hundred eighty-eight of 310 eligible patients were seen for follow-up at a median of 5.8 years after the initial assessment (Figure S1). Of the included patients, 150 had arterial stiffness assessments at baseline and follow-up, while 139 had TCD performed on both occasions. Demographic characteristics were similar between patients undergoing arterial stiffness and TCD measures (Table 1).

**Table 1.**
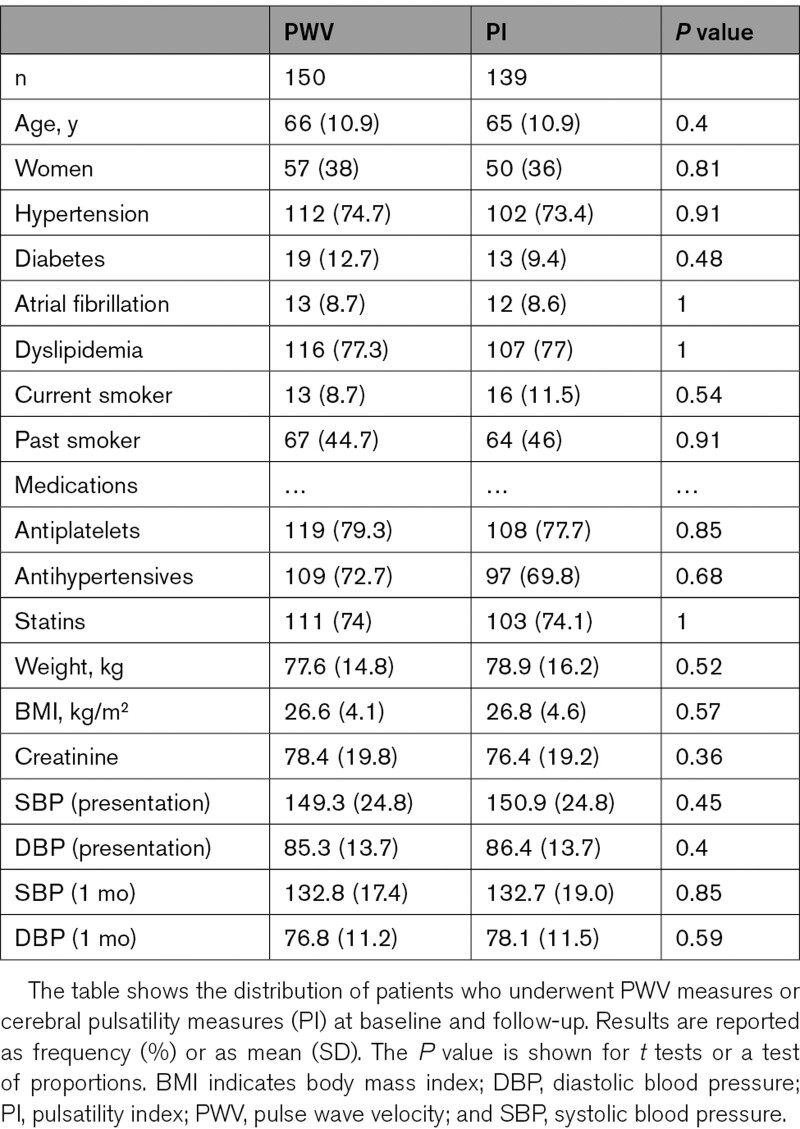
Demographics of Patients at Presentation With Repeat Studies After 5-Year Follow-Up

**Figure 1.**
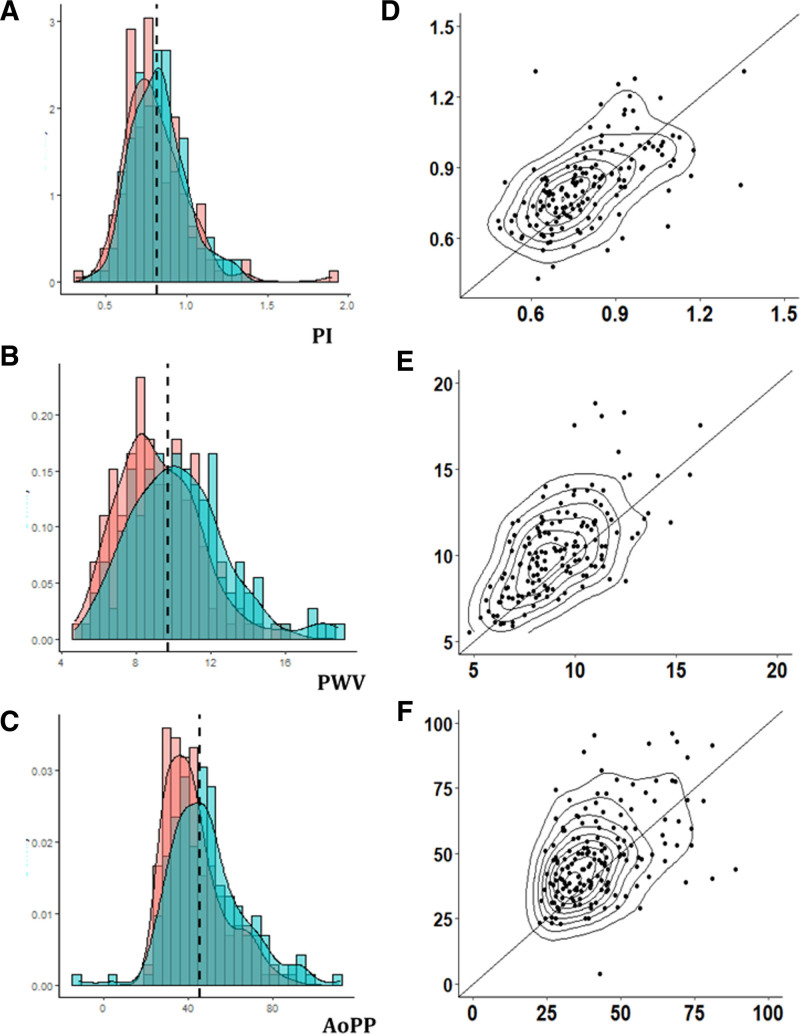
**Distribution and reproducibility of middle cerebral artery pulsatility index (PI), aortic pulse wave velocity (PWV), and aortic pulse pressure (aoPP). A–C**, Distributions for PI, PWV, and aoPP, respectively, for baseline vs follow-up. **D–F**, Scatter plots for baseline vs follow, including a line of unity.

Arterial stiffness (PWV), aoPP, and cerebral arterial pulsatility were all highly reproducible within individuals over the 5 years of follow-up (Table 2; Figure S2), with PWV as the most reproducible index. Absolute indices of aortic blood pressure or CBFV were also significantly reproducible, but less so than pulsatility or arterial stiffness measures, with weaker correlations between baseline and follow-up values (Table 2; Figure 1).

**Table 2.**
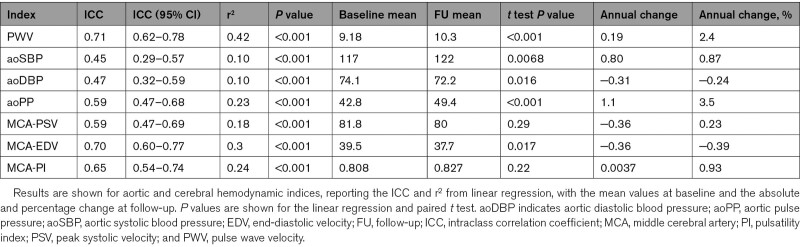
Reproducibility and Progression of Arterial Stiffness, Aortic Blood Pressure, and Cerebral Blood Flow Velocity

There were significant increases in arterial stiffness and aoPP between baseline and follow-up, with the greatest annual increase in aoPP (3.5% per year). Cerebral arterial pulsatility increased nonsignificantly across all patients (0.93%; Table 2) but was affected by a single outlier with a fall in PI of >4 SDs from the mean. In a post hoc sensitivity analysis, exclusion of this patient demonstrated significant overall increase (absolute change per annum, 0.005; 1.02%; *P*=0.04). The increase in aoPP reflected a significant fall in aoDBP (Table 2; Figure S3), with a rise in aoSBP (*P*=0.01), but both cerebral peak and trough flow velocities fell with time (Table 2).

**Figure 2.**
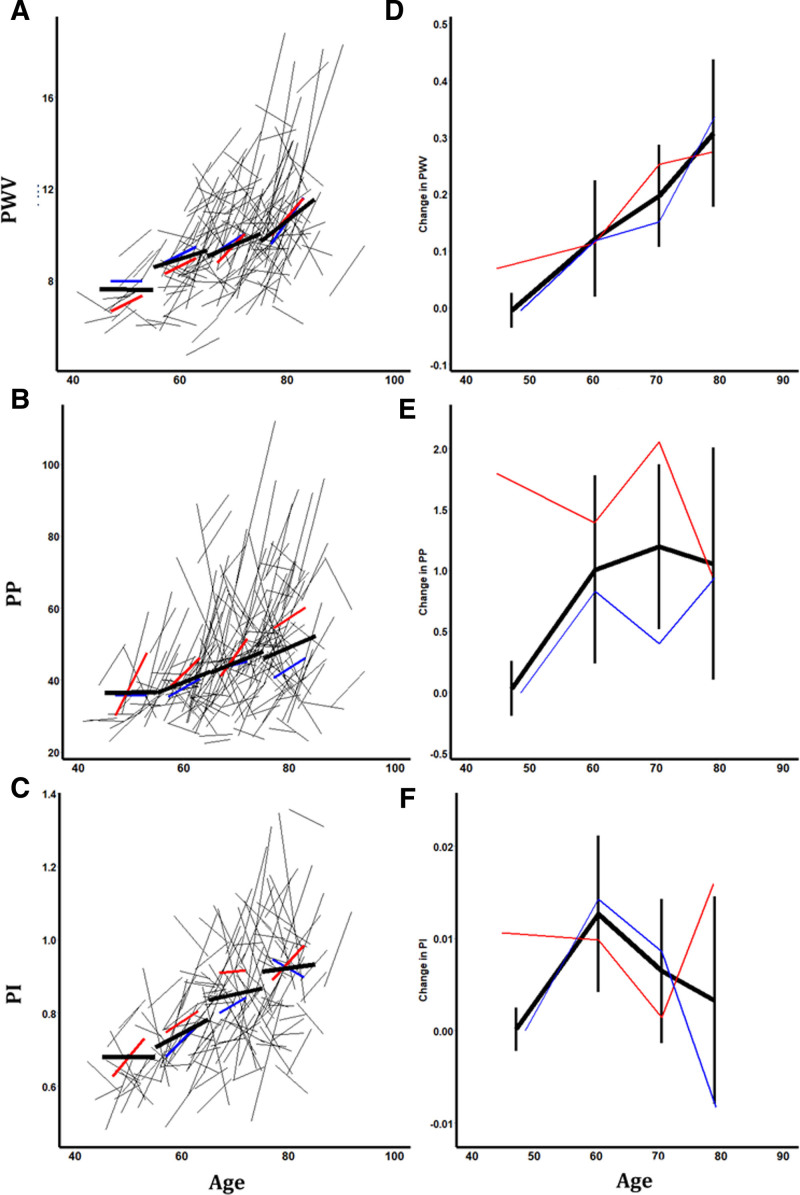
**Progression of aortic blood pressure and cerebral blood flow velocity by age and sex. A–C**, Individual changes during follow-up, and summary estimates within age groups (<55, 55–65, 65–75, and >75), for all patients (black), for men (blue) and women (red). **D–F**, Average rate of progression within each age group, stratified by age and sex. PI indicates pulsatility index; PP, pulse pressure; and PWV, pulse wave velocity.

Carotid-femoral PWV was higher at older ages, with no significant yearly increase in patients below the age of 55 years, but PWV increased at a greater annual rate with increasing age (Figure 2; Table 1). Although PWV was greater in men at each age, the rate of increase in PWV with age was similar in men and women (Figure 1). Similarly, both aoPP and cerebral arterial pulsatility increased at a greater rate above the age of 55 years with no significant increase before 55 years (>55 versus <55: aoPP, *P*=0; MCA-PI, *P*=0.009), although the rate of change of aoPP or MCA-pulse pressure above 55 years was not consistent across age groups (Figure 2).

aoPP was significantly greater in women than in men and increased at a faster rate (Table 3), while MCA-PI was nonsignificantly greater in women than in men with no difference in the rate of increase (Figure 1; Table 3). The rise in aortic and cerebral pulsatility at older ages was paralleled by a higher aoSBP with increasing age, with a higher rate of increase at older ages. In contrast, PSV and EDV were lower at older ages and were lower in men compared with women, while the rate of change in PSV and EDV was similar across ages (Table S1; Figure S2).

**Table 3.**
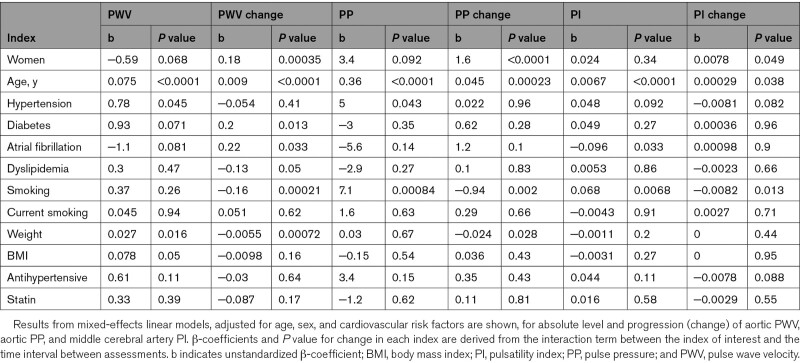
Associations Between Demographic Characteristics and Progression of Key Indices of Arterial Stiffness and Pulsatility

Elevated aoSBP, aoDBP, and aoPP were all associated with a greater rate of progression of PWV (Table 4), but cross-sectionally, higher PWV was associated with a lower concurrent DBP reflecting the decrease in DBP with time and age. After adjustment for heart rate and other aortic blood pressure values, only a higher aortic mean pressure was associated with a greater rate of increase in PWV with time (*P*=0.048). Similarly, higher aoSBP and aoDBP, elevated heart rate, and a greater cerebral pulsatility were associated with a greater rate of increase in aoPP, with persistent associations with progression of aoPP and aoSBP and aoDBP, even after adjustment for arterial stiffness and aortic blood pressure (*P*=0.0001 for both).

**Table 4.**
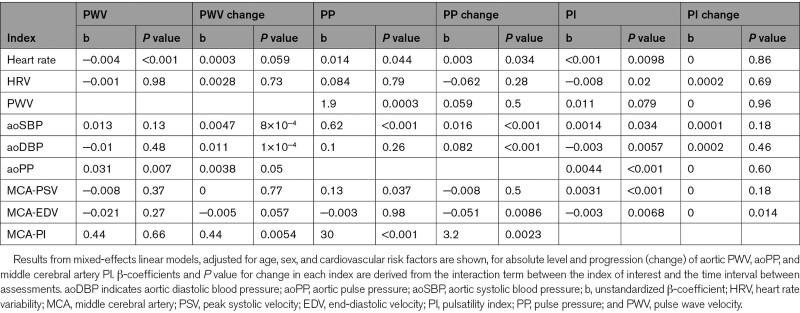
Associations Between Physiological Characteristics and Progression of Key Indices of Arterial Stiffness and Pulsatility

In contrast to aortic measures, a greater rate of progression of MCA-PI was only associated with increased age and female sex (Table 3) and a lower cerebral end-diastolic velocity (Table 4), with no association between blood pressure level or arterial stiffness with progression of MCA-PI before or after adjustment for age, sex, and cardiovascular risk factors. However, there were strong cross-sectional associations between MCA-PI and a concurrent high aoSBP, low aoDBP, and high aoPP.

## Discussion

This is the first study of progression of aortic stiffness, aoPP, and cerebral arterial pulsatility in a single patient cohort, following good blood pressure control. Over 5 years, aortic stiffness and aortic systolic pressure rose while aortic diastolic pressure fell, with a resulting increase in aoPP. Cerebral pulsatility rose above the age of 55 years, reflecting a fall in end-diastolic velocity with no rise in peak systolic velocity. The rate of rise in PWV and aoPP was associated with age and elevated aortic blood pressure, but only age, female sex, and a low end-diastolic flow velocity predicted an increased rate of rise in MCA-PI, although the current MCA-PI level was strongly associated with concurrent aoPP.

In previous cross-sectional analyses in this population,^7,17^ cerebral SVD was associated with cerebral arterial pulsatility, which was principally associated with aortic stiffness and transmission of increased aoPP to the brain. Furthermore, there were similar distributions of aortic stiffness, aoPP, and cerebral arterial pulsatility by age and sex, and the cross-sectional relationship between aoPP and cerebral arterial pulsatility was partially mediated by arterial stiffness.^19^ This longitudinal follow-up study confirms the strong concurrent association between aoPP, aortic stiffness, and cerebral arterial pulsatility and further demonstrates that aortic blood pressure level predicts a greater increase in the rate of progression of aortic stiffness and pulsatility. As such, although an acute reduction in aoPP may reduce cerebral arterial pulsatility directly, control of midlife elevated aortic blood pressure, and DBP in particular, may reduce progression of arterial stiffness and aoPP and, therefore, future cerebral SVD and its sequelae. Furthermore, this population had undergone intensive blood pressure monitoring and treatment, with a minimal difference between treatment at the time of initial assessment and at follow-up 5 years later,^20^ although there was an increase in clinic systolic blood pressure between baseline and follow-up, potentially influencing measurement of PWV, although this could reflect a white-coat response especially as home readings were not available. Nonetheless, this study demonstrates that residual aortic blood pressure and pulsatility were still associated with increased cerebral pulsatility despite excellent control of blood pressure and other risk factors, presenting a potentially modifiable risk factor in addition to current standard treatment.

The majority of the progression in arterial stiffness and pulsatility occurred after the age of 55 years, with steadily increasing rates of progression of arterial stiffness consistent with previous studies in hypertensive populations. Both aortic and cerebral pulsatility were greater above 55 years but with a limited ongoing increase in the rate of progression thereafter. This apparent transition at ≈55 years of age is consistent with the average age of transition to late-life hypertensive phenotypes^21^ and the stronger relationship between midlife elevated DBP with cerebral white matter hyperintensities,^22^ compared with greater systolic blood pressure and pulsatility above the age of 55 years. Furthermore, although DBP fell as arterial stiffness increased, a higher aoDBP was still the strongest driver of progression of PWV and, therefore, a subsequent fall in DBP. This indicates the likely critical role for control of midlife DBP to prevent later progression of aortic stiffness and pulsatility and resulting cerebral pulsatility.

Although no systemic hemodynamic measures were specifically associated with progression of cerebral pulsatility, current cerebral pulsatility was most strongly associated with concurrent aoPP. As such, targetting both excess aortic systolic pressure and diastolic pressure in midlife while not excessively reducing diastolic pressure in late life may reduce progression of aoPP and, therefore, later-life cerebral arterial pulsatility. This may be possible with peripheral vasodilators that reduce wave reflection from the peripheral circulation. For example, in the CAFE study (Conduit Artery Function Evaluation),^23^ amlodipine reduced aortic systolic pressure to a greater extent than a β-blocker, while cilostazol reduced cerebral pulsatility in the ECLIPse study (Effect of Cilostazol in Acute Lacunar Infarction Based on Pulsatility Index of Transcranial Doppler).^24^ Currently, isosorbide mononitrate and cilostazol are being assessed in the LACI (Lacunar Intervention)-1/LACI-2 studies (https://www.clinicaltrials.go; unique identifier: NCT03451591),^25^ antihypertensives in the TREAT-SVDs trial (Effects of Amlodipine & Other Blood Pressure Lowering Agents on Microvascular Function in Small Vessel Diseases; https://www.clinicaltrials.gov; unique identifier: NCT03082014), and sildenafil in the OxHARP trial (Oxford Haemodynamic Adaptation to Reduce Pulsatility; https://www.clinicaltrials.gov; unique identifier: NCT03855332).

This study does have some limitations. First, it is relatively small. However, it is still significantly larger than any other available study recording both aortic and cerebral indices, and other studies have not assessed progression of these measures in this patient population.^26,27^ Furthermore, despite the moderate size, associations were strong and consistent with previous evidence while building upon it. Second, not all participants underwent both TCD and arterial stiffness measures. This is inevitable in a pragmatic study including all patients with transient ischemic attack or minor stroke, due to the relatively high incidence of participants with poor bone windows for TCD or, for PWV, significant large artery disease or increased body mass index. Third, due to the age and frailty of the population, only 50% of the baseline population could be reassessed at 5 years, as many were deceased or had moved out of the area. This may result in underestimation of the degree of progression of age-related variables due to attrition of higher risk patients with greater progression, but any resulting bias is likely to be conservative. Furthermore, the median age of the population was relatively old for a follow-up study (mean age, 66 years at baseline and 71 years at follow-up), supporting the relevance of these results to the population most at risk. Fourth, patients with atrial fibrillation were included in the analyses as PWV and PI are still relevant in this group, but atrial fibrillation may reduce reliability of assessment of PWV in particular. Finally, without repeat brain imaging, we were unable to assess whether progression of cerebral arterial pulsatility mediates the relationship between arterial stiffness, aoPP, and progression of cerebral SVD.

Overall, the strong relationship between residual aortic blood pressure after intensive blood pressure treatment and longitudinal progression of arterial stiffness and pulsatility supports the need for further research to determine the temporal relationship with other key physiological mechanisms affected in SVD, such as blood pressure variability, cerebral autoregulation, and cerebrovascular reactivity, which represent alternative potential treatment targets. Further trials will then be necessary to determine whether modifying cerebral pulsatility or other mechanisms translates to a reduction in progression of cerebral SVD, proceeding to large clinical trials to determine whether such interventions translate to meaningful clinical effects.

## Conclusions

Arterial pulsatility and aortic stiffness progressed significantly over the age of 55 years in a population of patients with transient ischemic attack or minor stroke. Progression of aortic measures was most strongly associated with age and elevated aortic blood pressure, while only age, sex, and low diastolic CBFV predicted progression of cerebral arterial pulsatility. However, aoPP was the strongest association of concurrent cerebral pulsatility, identifying a potentially treatable mechanism to limit progression of cerebral SVD.

## Article Information

### Acknowledgments

We are grateful to all the staff in the general practices that collaborated in the OXVASC study (Oxford Vascular Study): Abingdon Surgery, Stert St, Abingdon; Malthouse Surgery, Abingdon; Marcham Road Family Health Centre, Abingdon; The Health Centre, Berinsfield; Key Medical Practice; Kidlington; 19 Beaumont St, Oxford; East Oxford Health Centre, Oxford; Church Street Practice, Wantage. This work uses data provided by patients and collected by the National Health Service as part of their care and support and would not have been possible without access to these data. The National Institute for Health Research recognizes and values the role of patient data, securely accessed and stored, both in underpinning and leading to improvements in research and care. This research was funded, in whole or in part, by the Wellcome Trust, grant number 206589/Z/17/Z. A CC BY licence is applied to author accepted manuscript arising from this submission, in accordance with the grant’s open access conditions. A.J.S. Webb devised, acquired, supervised, and analyzed the physiological assessments; devised, analyzed, and supervised the statistical analysis; and drafted, analyzed, edited, and submitted the manuscript. A. Lawson, Dr Mazzucco, and K. Wartolowska acquired data. A. Lawson analyzed and cleaned data. P.M. Rothwell established and supervised the OXVASC study; devised, initiated, and supervised the physiological studies and statistical analyses; and edited and supervised the manuscript.

### Sources of Funding

The OXVASC (Oxford Vascular Study) is funded by the National Institute for Health Research (NIHR) Oxford Biomedical Research Centre (BRC), Wellcome Trust, Wolfson Foundation, British Heart Foundation, and the European Union Horizon 2020 programme (grant 666881, SVDs@Target). P.M. Rothwell is in receipt of an NIHR Senior Investigator award. A.J.S. Webb and this work is funded by a Wellcome Trust Clinical Research Development Fellowship (206589/Z/17/Z) and British Heart Foundation Project grant (PG/16/38/32080). The views expressed are those of the authors and not necessarily those of the NHS, the NIHR, or the Department of Health.

### Disclosures

None.

### Supplemental Material

Figures S1–S3

Tables S1–S4

## Supplementary Material


