# Supplementary material for: Aortic Stiffness, Pulse Pressure, and Cerebral Pulsatility Progress Despite Best Medical Management: The OXVASC Cohort
Source: Stroke. 2021 Dec 2;53(4):1310–7. doi: 10.1161/STROKEAHA.121.035560 (PMC7612543; doi:10.1161/STROKEAHA.121.035560)
Supplement: Supplementary file 1 [file str-53-1310-s001.pdf]

SUPPLEMENTAL DATA

Aortic stiffness, pulse pressure and cerebral pulsatility progress despite best medical management: the OXVASC cohort

Alastair JS Webb, Amy Lawson, Karolina Wartolowska, Linxin Li, Sara Mazzucco, Peter M Rothwell

Supplemental Figure I. Flowchart of included patients

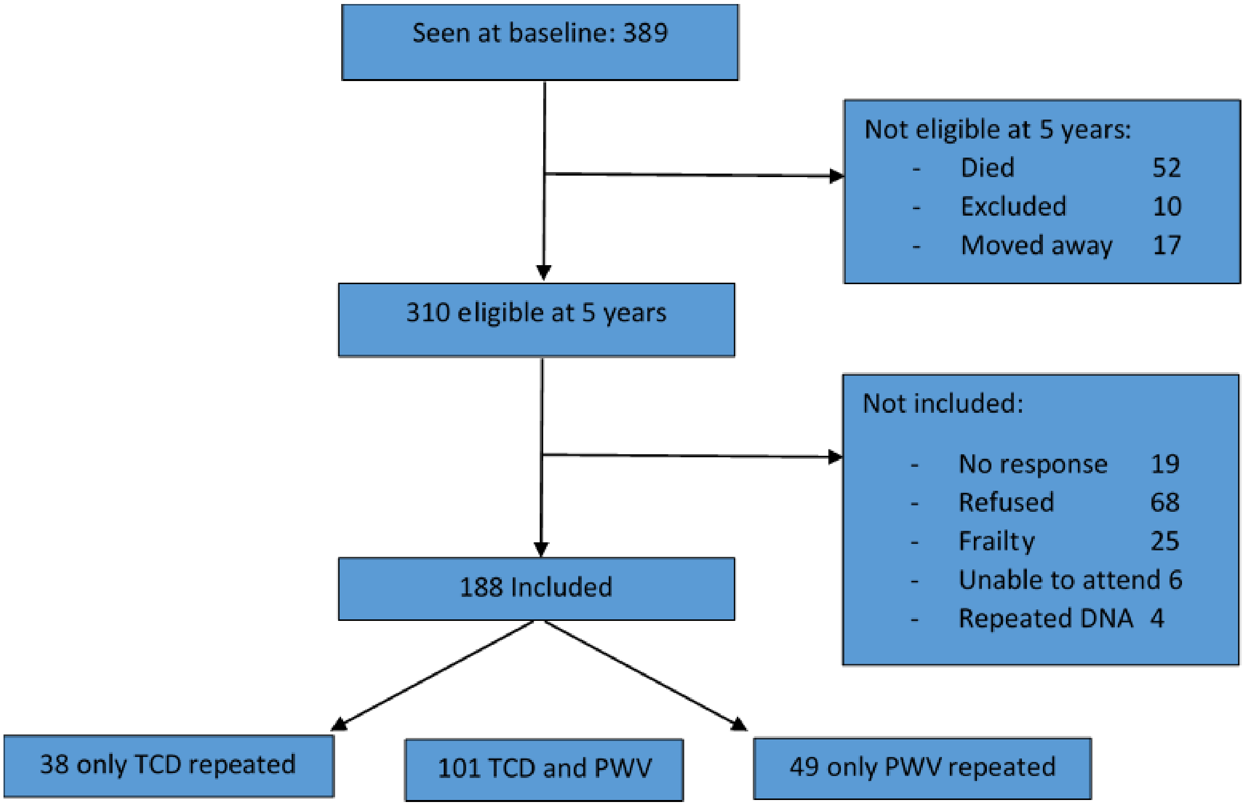

Supplemental Figure II. Bland-Altman plots for middle cerebral blood flow velocity (PI), Aortic pulse wave velocity (PWV) and aortic pulsatility (AoPP). Results are shown for the mean versus difference in measures for individuals from baseline to follow-up.

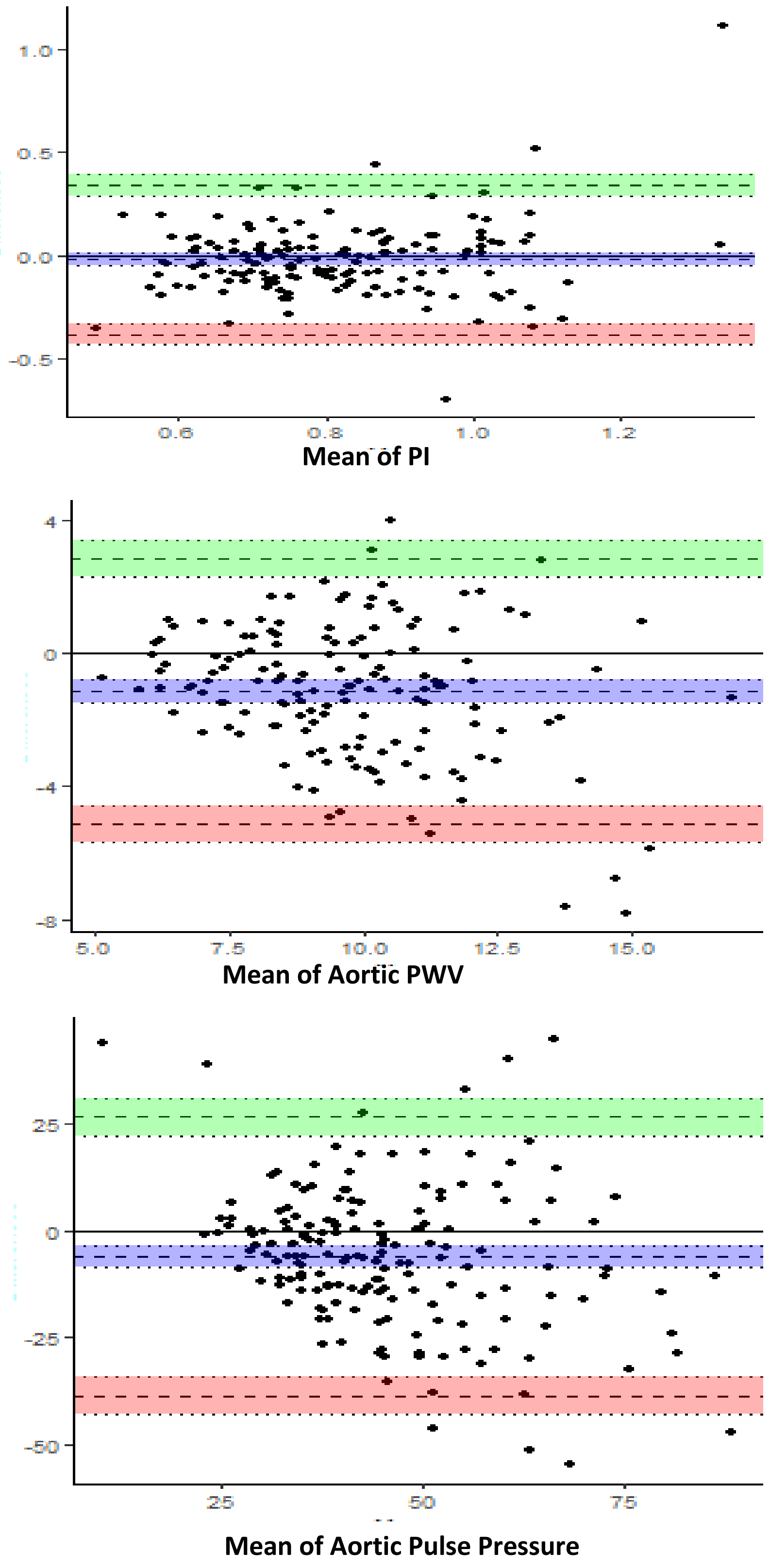

Supplemental Figure III. **Progression of aortic blood pressure and cerebral blood flow velocity by age and gender.** Panels A-D show individual changes during follow-up, and summary estimates within age groups (<55, 55-65,65-75, >75), for all patients (black), for men (blue) and women (red). Panels E-H show the average rate of progression within each age group, divided by age and gender

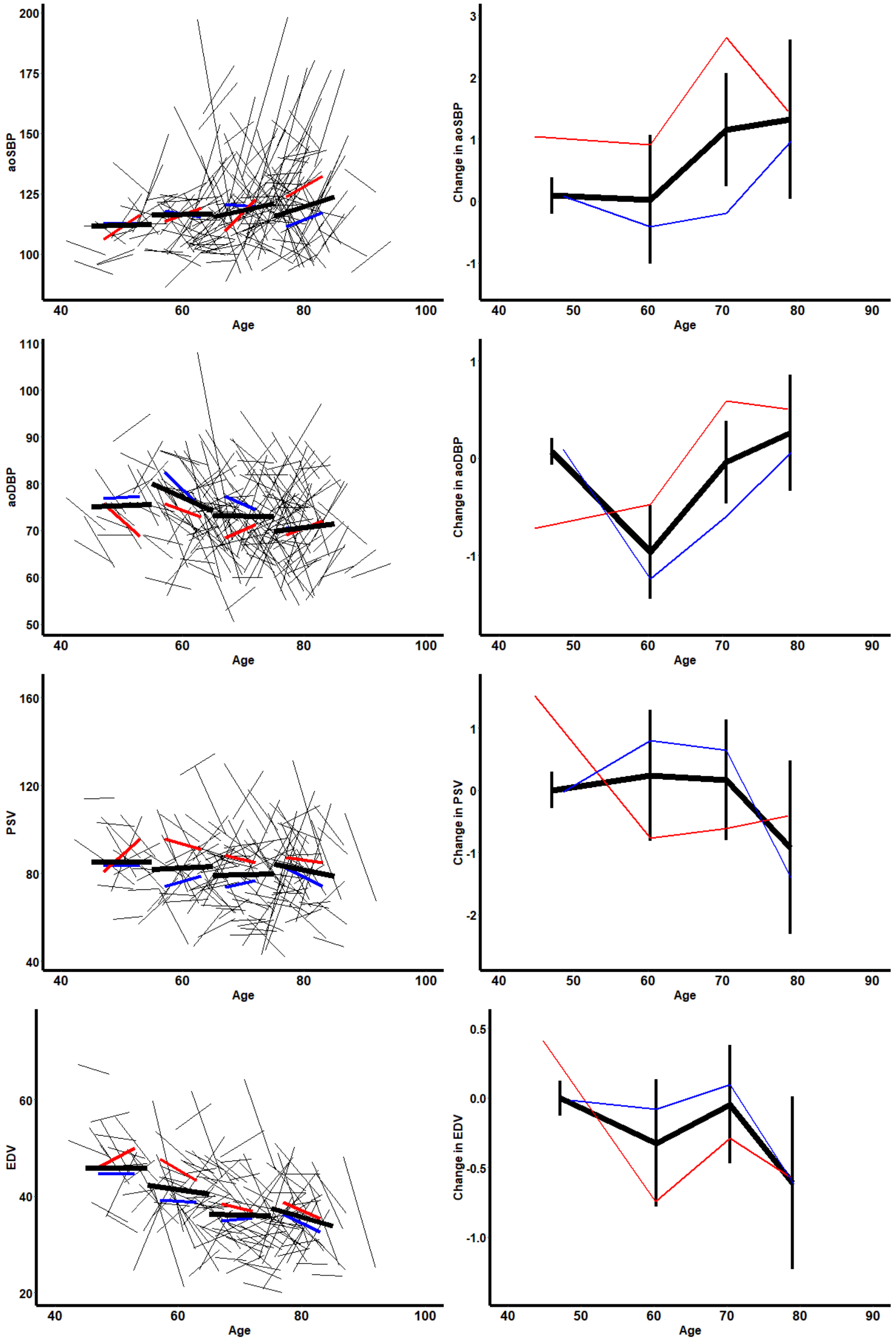

Supplemental Table I. Unadjusted asssociations between demographic measures and annual absolute progression of arterial stiffness, aortic pulsatility and cerebral pulsatility. Results from mixed effect linear models, unadjusted for covariates for absolute level and progression (change) of aortic pulse wave velocity (PWV), aortic pulse pressure (PP) and middle cerebral artery pulsatility index (PI). B = unstandardized beta-coefficient; p= p-value. Beta-coefficients and p-values for change in each index are derived from the interaction term between the index of interest and the time interval between assessments.

Index	PWV		PWV change		PP		PP change		PI		PI change	
	b	p	b	p	b	p	b	p	b	p	b	p
Female	-0.6	0.11	0.18	0.0003	3.7	0.077	1.6	<0.001	0.03	0.28	0.0064	0.11
Age	0.08	<0.001	0.009	<0.001	0.34	0.0002	0.045	0.0002	0.007	<0.001	0.0003	0.035
Hypertension	1.40	0.001	-0.038	0.56	7.7	0.0032	0.15	0.74	0.098	0.0022	-0.007	0.14
Diabetes	1.50	0.0076	0.2	0.012	-1.7	0.63	0.63	0.27	0.087	0.072	0.0008	0.92
Atrial Fibrillation	-0.82	0.23	0.22	0.032	-1.8	0.66	1.1	0.11	-0.041	0.42	-0.001	0.87
Dyslipidaemia	0.72	0.11	-0.13	0.052	-0.94	0.73	0.15	0.75	0.037	0.28	-0.001	0.87
Smoking	0.35	0.33	-0.15	<0.001	4.4	0.043	-0.85	0.0049	0.044	0.10	-0.008	0.017
Current Smoking	-0.97	0.13	0.057	0.58	-2.5	0.50	0.11	0.87	-0.062	0.17	-0.002	0.79
Weight	0.022	0.063	-0.005	0.0009	-0.11	0.10	-0.023	0.039	-0.002	0.024	0	0.43
BMI	0.072	0.11	-0.009	0.19	-0.32	0.22	0.039	0.41	-0.005	0.15	0	0.82
Antihypertensive	1.30	0.0027	-0.018	0.77	6.6	0.008	0.45	0.31	0.098	0.0015	-0.007	0.15
Statin	0.77	0.073	-0.089	0.16	1.2	0.64	0.17	0.71	0.048	0.14	-0.001	0.84

Supplemental Table II. Unadjusted asssociations between physiological measures and progression of arterial stiffness, aortic pulsatility and cerebral pulsatility. Unadjusted results from mixed effect linear models for absolute level and progression (change) of aortic pulse wave velocity (PWV), aortic pulse pressure (PP) and middle cerebral artery pulsatility index (PI). Beta-coefficients and p-values for change in each index are derived from the interaction term between the index of interest and the time interval between assessments. b = unstandardized beta-coefficient; p= p-value; SBP = systolic blood pressure; DBP =diastolic blood pressure; MCA = middle cerebral artery; PSV = peak systolic velocity; EDV = end-diastolic velocity; PI = pulsatility index.

Index	PWV		PWV change		PP		PP change		PI		PI change	
	b	p	b	p	b	p	b	p	b	p	b	p
Heart Rate	-0.0048	<0.001	0.0003	0.067	0.0081	0.27	0.0026	0.07	0.0002	0.034	0	0.84
HRV	-0.018	0.72	0.0064	0.44	-0.093	0.78	-0.015	0.8	-0.0096	0.0048	0.0004	0.47
PWV					2.2	<0.001	0.059	0.5	0.023	<0.001	-0.0001	0.92
Ao SBP	0.015	0.087	0.0049	0.0006	0.64	<0.001	0.016	0.0004	0.0018	0.0066	0.0001	0.2
Ao DBP	-0.023	0.13	0.011	<0.001	0.044	0.64	0.088	<0.001	-0.0035	0.0019	0.0002	0.47
Ao PP	0.041	0.0005	0.0035	0.081					0.0054	<0.001	<0.001	0.76
MCA PSV	-0.014	0.14	-0.0004	0.8	0.12	0.055	-0.011	0.36	0.0029	<0.001	-0.0002	0.14
MCA EDV	-0.054	0.0032	-0.0055	0.05	-0.11	0.34	-0.053	0.0071	-0.0052	4e-05	-0.0005	0.017
MCA PI	2.6	0.011	0.45	0.0062	36	<0.001	3.1	0.0037				

Supplemental Table III. Asssociation between demographic measures and progression of absolute measures of aortic blood pressure and cerebral blood flow velocity. Results from mixed effect linear models, unadjusted for covariates, for absolute level and progression (change) of aortic systolic (SBP) and diastolic (DBP) blood pressure and middle cerebral artery peak (PSV) and end-diastolic (EDV) flow velocity. B = unstandardized beta-coefficient; p= p-value. Beta-coefficients and p-values for change in each index are derived from the interaction term between the index of interest and the time interval between assessments.

Index	SBP		SBP Change		DBP		DBP Change		PSV		PSV change		EDV		EDV change	
	b	p	b	p	b	p	b	p	b	p	b	p	b	p	b	p
Female	1.1	0.67	1.8	<0.001	-2.3	0.072	0.18	0.4	10	<0.001	-0.43	0.38	4.1	0.002	-0.39	0.069
Age	0.29	0.01	0.041	0.011	-0.08	0.18	-0.004	0.58	-0.27	0.035	-0.007	0.67	-0.37	<0.001	-0.012	0.12
Hypertension	6	0.052	0.51	0.4	0.94	0.54	0.5	0.079	-0.85	0.8	0.22	0.7	-1.6	0.29	0.36	0.15
Diabetes	-3.7	0.36	0.11	0.88	-0.5	0.81	-0.54	0.14	15	0.006	-0.72	0.43	4.5	0.062	-0.097	0.81
AF	-2.5	0.6	1	0.27	3.1	0.2	-0.11	0.81	1.1	0.84	-0.92	0.35	3.2	0.19	-0.36	0.41
Dyslipidaemia	-7.8	0.018	0.8	0.21	-4.9	0.003	0.7	0.021	3	0.41	0.25	0.7	1.6	0.33	0.2	0.47
Smoking ever	3.8	0.15	-0.39	0.33	-3.3	0.013	0.55	0.004	0.009	1	-0.008	0.98	-2.1	0.14	0.28	0.11
Current Smoking	5.3	0.21	-0.79	0.36	3.7	0.081	-1.1	0.009	0.021	1	0.31	0.73	-0.14	0.95	0.14	0.72
Weight	0.082	0.34	-0.03	0.037	0.05	0.24	-0.006	0.41	-0.13	0.18	0.033	0.03	-0.03	0.5	0.018	0.009
BMI	0.006	0.98	-0.004	0.94	0.15	0.33	-0.039	0.17	-0.27	0.42	0.11	0.083	-0.02	0.89	0.043	0.12
Antihypertensive	3.9	0.19	1	0.075	0.37	0.8	0.7	0.013	-1.9	0.56	0.18	0.75	-2	0.19	0.35	0.15
Statin	-5.5	0.071	0.96	0.1	-4.2	0.006	0.85	0.002	2.6	0.46	-0.053	0.93	1.1	0.49	0.079	0.76

Supplemental Table IV. Asssociation between physiological measures and progression of absolute measures of aortic blood pressure and cerebral blood flow velocity. Results from mixed effect linear models, unadjusted for covariates, for absolute level and progression (change) of aortic systolic (SBP) and diastolic (DBP) blood pressure and middle cerebral artery peak (PSV) and end-diastolic (EDV) flow velocity. B = unstandardized beta-coefficient; p= p-value. Beta-coefficients and p-values for change in each index are derived from the interaction term between the index of interest and the time interval between assessments.

Index	SBP		SBP Change		DBP		DBP Change		PSV		PSV change		EDV		EDV change	
	b	p	b	p	b	p	b	p	b	p	b	p	b	p	b	p
Heart Rate	0.0074	0.4	0.0017	0.38	-0.007	0.14	-0.001	0.13	0.012	0.2	-0.001	0.46	-0.001	0.79	0	0.35
HRV	0.24	0.56	-0.052	0.49	0.15	0.46	0.017	0.65	-0.61	0.13	-0.029	0.69	-0.085	0.63	0.0028	0.94
PWV	2.5	0.0002	-0.021	0.85	0.64	0.051	-0.084	0.14	-0.76	0.31	0.037	0.78	-0.63	0.052	0.016	0.77
Ao SBP					0.38	<0.001	-0.016	<0.001	0.034	0.68	<0.001	0.97	-0.032	0.38	-0.004	0.49
Ao DBP	1.1	<0.001	0.082	<0.001					-0.28	0.042	-0.009	0.72	-0.027	0.67	-0.006	0.62
Ao PP	1.1	<0.001	0.011	0.21	0.071	0.14	0.011	0.21	0.21	0.058	-0.009	0.65	-0.036	0.47	-0.007	0.44
MCA PSV	0.034	0.67	-0.001	0.95	-0.1	0.011	0.0068	0.38					0.35	<0.001	0.0046	0.26
MCA EDV	-0.14	0.36	-0.029	0.25	-0.15	0.059	0.022	0.094	1.9	<0.001	-0.027	0.065				
MCA PI	20	0.027	3.1	0.029	-9.7	0.032	-0.1	0.89	43	<0.001	-1.6	0.22	-12	0.0022	-0.75	0.22

STROBE Statement—checklist of items that should be included in reports of observational studies

	Item No	Recommendation	Page
**Title and abstract**	1	(*a*) Indicate the study's design with a commonly used term in the title or the abstract	1
		(*b*) Provide in the abstract an informative and balanced summary of what was done and what was found	2
**Introduction**			
Background/rationale	2	Explain the scientific background and rationale for the investigation being reported	5
Objectives	3	State specific objectives, including any prespecified hypotheses	5
**Methods**			
Study design	4	Present key elements of study design early in the paper	7
Setting	5	Describe the setting, locations, and relevant dates, including periods of recruitment, exposure, follow-up, and data collection	7
Participants	6	(*a*) *Cohort study*—Give the eligibility criteria, and the sources and methods of selection of participants. Describe methods of follow-up<br>*Case-control study*—Give the eligibility criteria, and the sources and methods of case ascertainment and control selection. Give the rationale for the choice of cases and controls<br>*Cross-sectional study*—Give the eligibility criteria, and the sources and methods of selection of participants	7
		(*b*) *Cohort study*—For matched studies, give matching criteria and number of exposed and unexposed<br>*Case-control study*—For matched studies, give matching criteria and the number of controls per case	N/A
Variables	7	Clearly define all outcomes, exposures, predictors, potential confounders, and effect modifiers. Give diagnostic criteria, if applicable	7-8
Data sources/ measurement	8*	For each variable of interest, give sources of data and details of methods of assessment (measurement). Describe comparability of assessment methods if there is more than one group	7-8
Bias	9	Describe any efforts to address potential sources of bias	8
Study size	10	Explain how the study size was arrived at	NA
Quantitative variables	11	Explain how quantitative variables were handled in the analyses. If applicable, describe which groupings were chosen and why	8
Statistical methods	12	(*a*) Describe all statistical methods, including those used to control for confounding	8
		(*b*) Describe any methods used to examine subgroups and interactions	8
		(*c*) Explain how missing data were addressed	7
		(*d*) *Cohort study*—If applicable, explain how loss to follow-up was addressed<br>*Case-control study*—If applicable, explain how matching of cases and controls was addressed<br>*Cross-sectional study*—If applicable, describe analytical methods taking account of sampling strategy	7
		(*e*) Describe any sensitivity analyses	8

Continued on next page

**Results**			
Participants	13*	(a) Report numbers of individuals at each stage of study—eg numbers potentially eligible, examined for eligibility, confirmed eligible, included in the study, completing follow-up, and analysed	9, S1
		(b) Give reasons for non-participation at each stage	9, S1
		(c) Consider use of a flow diagram	S1
Descriptive data	14*	(a) Give characteristics of study participants (eg demographic, clinical, social) and information on exposures and potential confounders	T1
		(b) Indicate number of participants with missing data for each variable of interest	T1
		(c) *Cohort study*—Summarise follow-up time (eg, average and total amount)	9
Outcome data	15*	*Cohort study*—Report numbers of outcome events or summary measures over time	9
		Case-control study—Report numbers in each exposure category, or summary measures of exposure	
		Cross-sectional study—Report numbers of outcome events or summary measures	
Main results	16	(*a*) Give unadjusted estimates and, if applicable, confounder-adjusted estimates and their precision (eg, 95% confidence interval). Make clear which confounders were adjusted for and why they were included	T3-4
		(*b*) Report category boundaries when continuous variables were categorized	T3-4
		(*c*) If relevant, consider translating estimates of relative risk into absolute risk for a meaningful time period	NA
Other analyses	17	Report other analyses done—eg analyses of subgroups and interactions, and sensitivity analyses	11
**Discussion**			
Key results	18	Summarise key results with reference to study objectives	12
Limitations	19	Discuss limitations of the study, taking into account sources of potential bias or imprecision. Discuss both direction and magnitude of any potential bias	15
Interpretation	20	Give a cautious overall interpretation of results considering objectives, limitations, multiplicity of analyses, results from similar studies, and other relevant evidence	16
Generalisability	21	Discuss the generalisability (external validity) of the study results	16
**Other information**			
Funding	22	Give the source of funding and the role of the funders for the present study and, if applicable, for the original study on which the present article is based	18

*Give information separately for cases and controls in case-control studies and, if applicable, for exposed and unexposed groups in cohort and cross-sectional studies.

Note: An Explanation and Elaboration article discusses each checklist item and gives methodological background and published examples of transparent reporting. The STROBE checklist is best used in conjunction with this article (freely available on the Web sites of PLoS Medicine at http://www.plosmedicine.org/, Annals of Internal Medicine at http://www.annals.org/, and Epidemiology at http://www.epidem.com/). Information on the STROBE Initiative is available at www.strobe-statement.org.
